# Supplementary material for: Phytostimulatory Influence of Comamonas testosteroni and Silver Nanoparticles on Linum usitatissimum L. under Salinity Stress
Source: Plants (Basel). 2021 Apr 16;10(4):790. doi: 10.3390/plants10040790 (PMC8073072; doi:10.3390/plants10040790)
Supplement: Supplementary file 1 [file plants-10-00790-s001.zip › plants-1142463-supplementary.pdf]

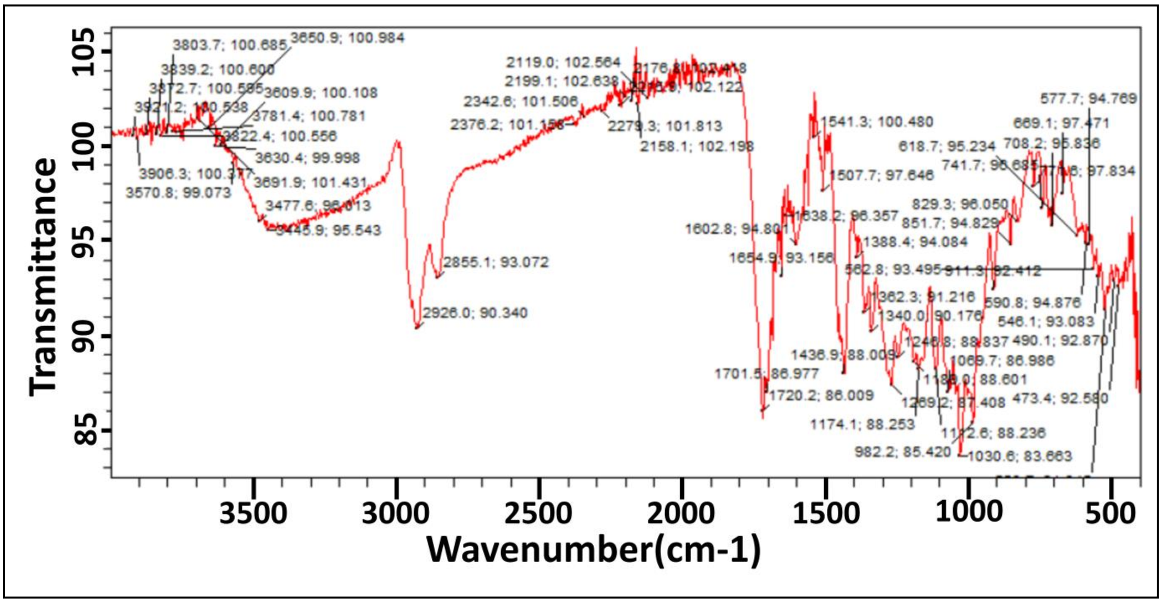

Figure S1. FT-IR spectrum of bio-synthesized AgNPs by *Rosmarinus officinalis* L. plants extract. The apparent peaks at 3570–3050, 2926–2856, 1720, 1602, 1602, 1436, 1340, 1269, 1174, 982, and 690–540 cm^{-1} were related to the specific functional groups. (Alcoholic compounds in the range ~3570–3050 cm^{-1} , 2926–2856 cm^{-1} extending the C-H bond's vibration corresponding to methylene, prominent peaks at 1720 and 1602 cm^{-1} could be attributed to extending the vibration of C=O that is allocated to aldehyde or ketone, 1602–1436–1340–1269 cm^{-1} attributed to stretching vibration of C=C bond referred to the aromatic compound. The 1174 cm^{-1} might be because of the stretching vibration of the aliphatic ether. The 1030 cm^{-1} might be because of the stretching vibration of S=O assigned to sulfoxide. At 982 cm^{-1}, a strong C=C bending vibration could be ascribed to a monosubstituted alkene. Strong peaks at 690–540 cm^{-1} may result from the stretching halo compound).

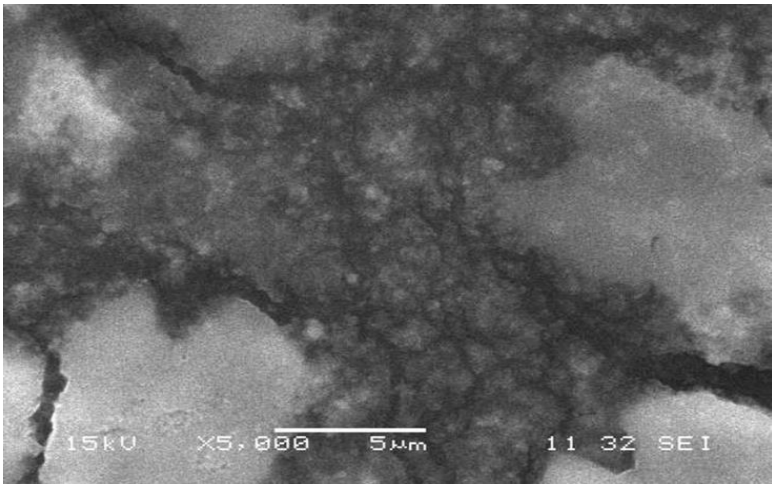

Figure S2. SEM micrograph of AgNPs bio-synthesized by *Rosmarinus officinalis* L. plants extract. Ag-nanoparticles are cuboid with approximately grain diameter of 75 nm,
